# Results of surgery for chronic pulmonary Aspergillosis, optimal antifungal therapy and proposed high risk factors for recurrence - a National Centre’s experience

**DOI:** 10.1186/1749-8090-8-180

**Published:** 2013-08-05

**Authors:** Shakil Farid, Shaza Mohamed, Mohan Devbhandari, Matthew Kneale, Malcolm Richardson, Sing Y Soon, Mark T Jones, Piotr Krysiak, Rajesh Shah, David W Denning, Kandadai Rammohan

**Affiliations:** 1 Department of Thoracic Surgery, University Hospital of South Manchester, Manchester, UK; 2 The National Aspergillosis Centre, University Hospital of South Manchester, The University of Manchester, Manchester Academic Health Science Centre, Manchester, UK

**Keywords:** Aspergilloma, Nodule, *Aspergillus fumigatus*, Voriconazole, Echinocandin

## Abstract

**Background:**

Surgery for pulmonary aspergillosis is infrequent and often challenging. Risk assessment is imprecise and new antifungals may ameliorate some surgical risks. We evaluated the medical and surgical management of these patients, including perioperative and postoperative antifungal therapy.

**Methods:**

Retrospective study of patients who underwent surgery for pulmonary aspergillosis between September 1996 and September 2011.

**Results:**

30 patients underwent surgery with 23 having a preoperative tissue diagnosis while 7 were confirmed post-resection. The median age was 57 years (17–78). The commonest presenting symptoms were cough (40%, n = 12) and haemoptysis (43%, n = 13). Twelve (40%) patients had simple aspergilloma (including 2 with *Aspergillus* nodules) while the remaining 18 (60%) had chronic cavitary pulmonary aspergillosis (CCPA) (complex aspergilloma). Most of the patients had underlying lung disease: tuberculosis (20%, n = 6), asthma (26%, n = 8) and COPD (20%, n = 6). The procedures included lobectomy 50% (n = 15), pneumonectomy 10% (n = 3), sublobar resection 27% (n = 8), decortication 7% (n = 2), segmentectomy 3% (n = 1), thoracoplasty 3% (n = 1), bullectomy and pleurectomy 3% (n = 1), 6% (n = 2) lung transplantation for associated disease. Median hospital stay was 9.5 days (3–37). There was no operative and 30 day mortality. Main complications were prolonged air leak (n = 7, 23%), empyema (n = 6, 20%), respiratory failure requiring tracheostomy /reintubation (n = 4, 13%). Recurrence of CCPA was noted in 8 patients (26%), most having prior CCPA (75%). Taurolidine 2% was active against all 9 *A. fumigatus* isolates and used for pleural decontamination during surgery.

**Conclusions:**

Surgery in patients with chronic pulmonary aspergillosis offered good outcomes with an acceptable morbidity in a difficult clinical situation; recurrence is problematic.

## Background

**Figure 1:**
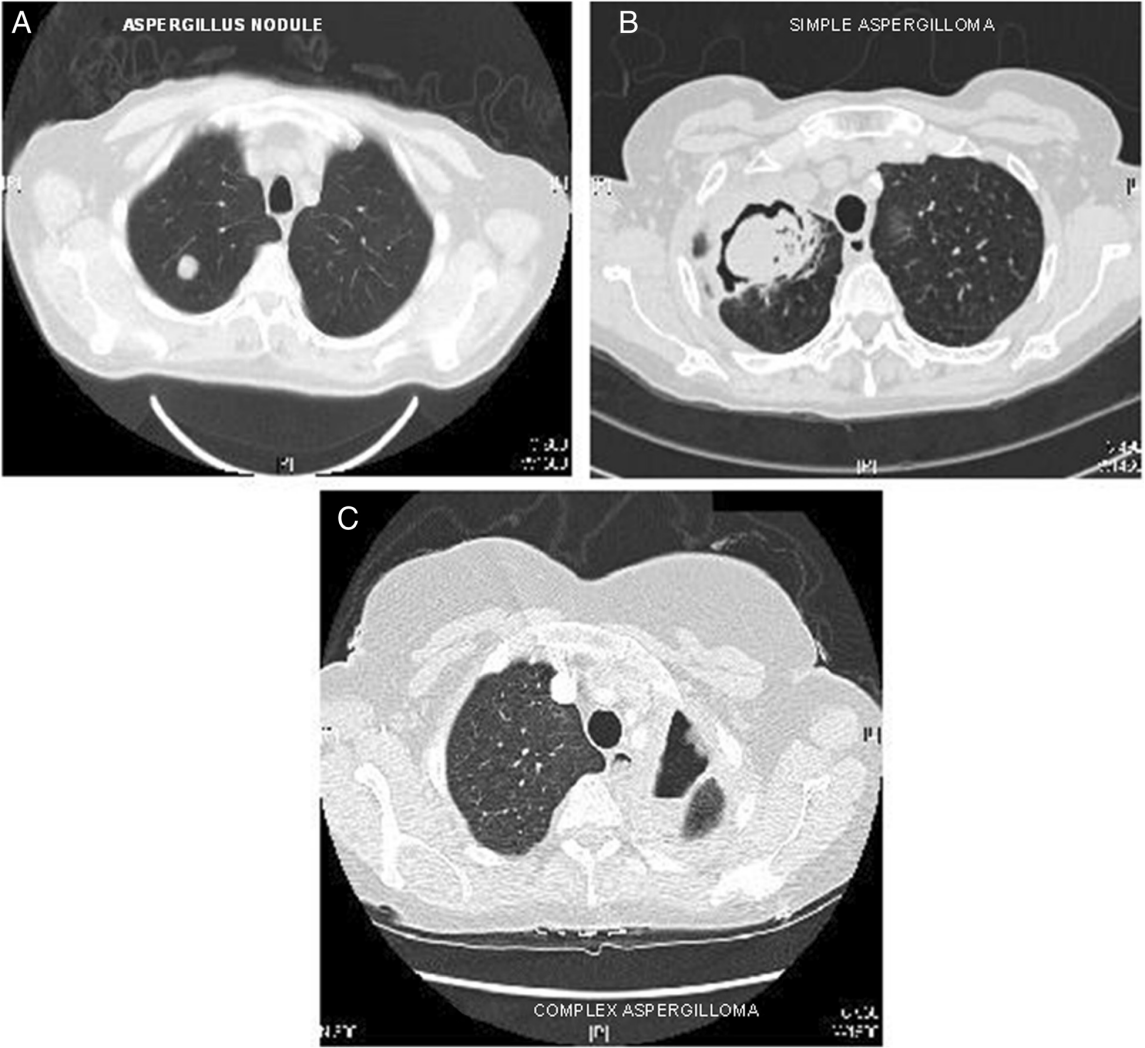
A: Examples of different types of chronic pulmonary aspergillosis aspergillus nodule; B: Simple aspergilloma; C: Chronic cavitary pulmonary aspergillosis (CCPA).

Aspergillosis refers to a spectrum of disease caused by *Aspergillus* species. This spectrum includes patients with asthma or cystic fibrosis who have allergic bronchopulmonary aspergillosis (ABPA) which is thought to affect over 4 million people worldwide [1]. In immunocompromised and critically ill patients invasive pulmonary aspergillosis (IPA) is relatively common and often fatal, and estimated to affect over 200,000 people worldwide [2]. In non-immunocompromised patients chronic pulmonary aspergillosis (CPA) (including those with an aspergilloma) may occur in those who have suffered a pulmonary insult such as tuberculosis, sarcoidosis, pneumothorax etc. [3]. The older term chronic necrotizing pulmonary aspergillosis mostly refers to those with subacute IPA. Within the spectrum of CPA are simple aspergilloma, chronic cavitary pulmonary aspergillosis, chronic fibrosing pulmonary aspergillosis and *Aspergillus* nodule [3,4] (Table 1) (Figure 1). There are estimated to be about 1.2 million cases of CPA following TB [5], perhaps 33-50% of the global total of patients with CPA.

**Table 1:** Definitions of chronic pulmonary aspergillosis and its subtypes

**Term**	**Definition**
Chronic pulmonary aspergillosis	Nodular or cavitary lesion or lesions in the lung, of at least 3 months duration in a non-immunocompromised patient (or one whose immunocompromising condition has remitted or is trivial), caused by *Aspergillus* spp. as demonstrated on tissue section by staining, by positive culture of a percutaneous biopsy or positive *Aspergillus* IgG antibodies.
Aspergilloma (Fungal ball caused by *Aspergillus* spp.)	An approximately spherical shadow with surrounding air, also called a fungal ball, in a pulmonary cavity, with serological or microbiological evidence that *Aspergillus* spp. is present in the material. This is a radiological or morphological description, not a disease descriptor and is not required for the diagnosis of chronic pulmonary aspergillosis.
Simple aspergilloma	Single pulmonary cavity containing a fungal ball, with serological or microbiological evidence implicating *Aspergillus* spp. in a non-immunocompromised patient with minor or no symptoms and no radiological progression over at least 3 months of observation.
Aspergillus nodule	One or more nodules which may or may not cavitate are an unusual form of CPA. They may mimic carcinoma of the lung or coccidioidomycosis and can only be definitively diagnosed on histology. Tissue invasion is not demonstrated, although necrosis is frequent.
Chronic cavitary pulmonary aspergillosis (CCPA)	One or more pulmonary cavities possibly containing an aspergilloma, with serological or microbiological evidence implicating *Aspergillus* spp. with significant pulmonary or systemic symptoms and overt radiological progression (new cavities, increasing pericavity infiltrates or increasing fibrosis) over at least 3 months of observation.
Chronic fibrosing pulmonary aspergillosis (CFPA)	Severe fibrotic destruction of at least two lobes of lung complicating CCPA leading to a major loss of lung function. Severe fibrotic destruction of one lobe with a cavity is simply referred to as CCPA affecting that lobe. Usually the fibrosis is manifest as consolidation, but large cavities with surrounding fibrosis may be seen.
Subacute invasive aspergillosis (SAIA) or chronic necrotising pulmonary aspergillosis (CNPA) (considered the same entity)	Invasive aspergillosis, usually in mildly immunocompromised patients, occurring over 1–3 months, with variable radiological features including cavitation, nodules, progressive consolidation with ‘abscess formation’. Biopsy shows hyphae in invading lung tissue and microbiological investigations reflect those in invasive aspergillosis, notably positive *Aspergillus* galactomannan antigen in blood (or respiratory fluids).

Controversy remains regarding the surgical management of both IPA and CPA in view of potential high mortality and morbidity. Surgery has been reserved for patients with unilateral, localized disease in IPA where curative resection is possible, as well as in patients with infection abutting the main pulmonary vessels to avoid fatal haemoptysis [6,7].

Surgical treatment is the mainstay of management for patients with simple aspergilloma [8]. Surgical results are excellent [8-10]. However there are many patients with extensive multicavity CPA who fail medical therapy and in whom surgery is contemplated. The results of surgical treatment for this group are not so good [11]. The perioperative management of post-surgical patients with pulmonary aspergillosis can be challenging.

Another dilemma is the duration of postoperative antifungal therapy in patients with an incidental diagnosis of aspergillosis. In 2011, we described a group of patients with PET positive lesions that were excised and found to contain *Aspergillus*-like hyphae on histology and positive *Aspergillus* IgG antibodies [4]. We have called these *Aspergillus* nodules; some are cavitating, others not, some single, many multiple. Given that some of these patients have more than one lesion and they all had risk factors for carcinoma of the lung, biopsy or surgical resection is inevitable. However their post-operative management and risk of recurrence is not described.

The objectives of our study were to analyze the short and long term outcomes of the surgical treatment for CPA including recurrence rates, evaluate the perioperative antifungal therapy, and formulate an algorithm for the follow-up of these patients including the further treatment of patients with an incidental diagnosis of aspergillosis.

## Methods

We examined the pre-operative findings and surgical indications, surgical procedure, histological findings and both short and long term outcomes of 30 patients who underwent surgery for pulmonary aspergillosis between September 1996 and September 2011. The Thoracic Surgery Department is co-located with the National Aspergillosis Centre (NAC) at the University Hospital of South Manchester. About 1,700 surgical procedures are done every year of which one fifth was major lung resections. The NAC manages over 250 patients with chronic pulmonary aspergillosis, with about 75 new referrals annually.

Patients with CPA and aspergilloma were classified into simple and complex aspergilloma according to previous classification by Belcher and Plummer [12,13], although we have used contemporary terminology for complex aspergilloma (chronic cavitary pulmonary aspergillosis (CCPA)). In this classification the simple aspergilloma cases had simple, isolated thin walled cysts or cavities lined by ciliated columnar epithelium, sometimes with overlying pleural thickening, and the surrounding lung was normal (Table 1). CCPA cases were defined as those with cavities developing within grossly diseased lung tissue, usually with extensive pleural thickening and lung contraction. Some may have had chronic fibrosing pulmonary aspergillosis (Table 1).

Diagnosis and classification of CPA was made based on clinical symptoms and radiological findings on CT scans and chest radiographs. The patients had preoperative fibreoptic bronchoscopy with sputum culture. CT guided percutaneous biopsy was done in cases of indeterminate lung nodules to rule out malignancy.

Medical records of all the patients were evaluated to collect patient characteristics, operation protocols, perioperative mortality and morbidity, histopathological findings, follow up information and short and long-term outcomes. Evaluation of perioperative antifungal therapy was also noted.

All pre-operatively diagnosed cases were discussed in the local specialist aspergillosis multidisciplinary meeting. Indications for surgery included unilateral localised disease where curative resection was possible, for symptom control, to manage complications, and for diagnosis of indeterminate lung nodule. Postoperatively the patients were followed up by the physicians at the National Aspergillosis Centre, to supervise antifungal therapy and determine duration of therapy and possible relapse.

### Microbiological method

Using previously published microtitre methodology [14] the minimum inhibitory concentrations (MIC) of taurolidine (Taurolin®) (molecular weight 284.36) was obtained by successive inoculation with solution into each microtitre well from 250 (0.5%) to 25 (0.05%) mg/L. Nine clinical isolates of *A. fumigatus* were tested, 2 of which were pan-azole resistant. Reading was a visual no growth endpoint at 48 hours after incubation at 37°C.

### Statistical analysis

IBM SPSS 20 software was used for statistical analysis. A P value of <0.05 was considered to be significant. Comparisons between patients were made by unpaired Student’s t-test for continuous variables and by Fisher’s exact test or chi square test for categorical variables. For survival analysis, Kaplan-Meier’s survival curve was used. Parametric variables were expressed with a median and binary variables were expressed with frequency.

## Results

Thirty patients underwent surgery with 23 (77%) having a preoperative tissue diagnosis while 7 (23%) were confirmed post resection. There were an equal number of males and females. The median age was 57 years (range 17–78). The major indications for surgery included recurrent haemoptysis (43%, n = 13), chronic cavitating lesions (26%, n = 8), lung nodules (23%, n = 7) and empyema (10%, n = 3). One patient with a simple aspergilloma had previously declined surgery, received long term antifungal therapy and grown pan-azole resistant *A. fumigatus*.

The commonest presenting symptoms amongst the 30 patients were cough (40%, n = 12) and haemoptysis (43%, n = 13) (Table 2). Among the 30 patients 12 (40%) had simple aspergilloma (Group 1) while the remaining 18 (60%) patients (Group 2) had CCPA. Most of the patients had underlying lung disease. Underlying lung diseases included tuberculosis (20%, n = 6), asthma (26%, n = 8), COPD (20%, n = 6), bronchiectasis (10%, n = 3), sarcoidosis (3%, n = 1) and cystic fibrosis (3%, n = 1). Six patients (20%) were immunosuppressed. Three patients had postoperative immunosuppression therapy because of transplantation or other associated disease, one patient had immunoglobulin deficiency, one patient had azathioprine for associated autoimmune hepatitis, and another had chemotherapy for associated lung cancer.

**Table 2:** Presenting symptoms of the 30 patients

	**GROUP 1**	**GROUP 2**	**P VALUE**
	**(N = 12)**	**(N = 18)**	
	**Simple aspergilloma**	**CCPA**	
Age	58 (27–78)	53.5 (17–77)	0.27
Male sex	7 (78%)	8 (42%)	0.7
Cough	5 (42%)	7 (38%)	1
Haemoptysis	6 (50%)	7 (38%)	0.71
Recurrent chest infections	6 (50%)	8 (44%)	1
Shortness of breath	4 (33%)	6 (33%)	1
Chest pain	29 (16%)	2 (11%)	1
Asymptomatic	3 (25%)	1 (5.5%)	0.27

The procedures included lobectomy 50% (n = 15), pneumonectomy 10% (n = 3), sublobar resection 26.6% (n = 8), decortication 6.6% (n = 2), segmentectomy 3% (n = 1), bullectomy and pleurectomy 3% (n = 1), thoracoplasty 3% (n = 1) (Table 3). Two patients (6%) underwent lung transplantation for associated cystic fibrosis and emphysema.

**Table 3:** Surgical procedures performed in aspergillosis

	**Group 1 (Simple)**	**Group 2 (CCPA)**	**P Value**
	**(N = 12)**	**(N = 18)**	
Lobectomy	8 (66.6%)	7 (38.8%)	0.26
Wedge resection	3 (25%)	5 (27.7%)	1
Segmentectomy	1 (8.3%)	0	0.4
Bullectomy and Pleurectomy	1 (8.3%)	0	0.4
Thoracoplasty	0	1 (5.5%)	1
Decortication	0	2 (11.1%)	0.5
Pneumonectomy	0	3 (16.6%)	0.25
Lung transplantation	0	2 (11.1%)	0.5

Median hospital stay was 9.5 days (3–37). Prolonged air leak (n = 7, 23%), empyema (n = 6, 20%), reopening for bleeding (n = 1, 3%), ARDS (n = 1, 3%), respiratory failure requiring tracheostomy/reintubation (n = 4, 13%) and bronchopleural fistula (n = 1, 3%) were the noted complications. Complications were more common in the CCPA patients (group 2).

Among the 30 patients 23 had preoperative diagnosis of aspergillosis and were on antifungal therapy. The remaining 7 had an incidental diagnosis of aspergillosis following surgery and received postoperative antifungal therapy. Two patients had single *Aspergillus* nodules, and histopathological examination showed necrosis surrounded by granulomatous inflammation with scattered multinuceate giant cells. The centre of the necrotic material revealed numerous fungal hyphae and, in one case, conidial heads with features in keeping with *Aspergillus*. The surrounding lung parenchyma showed fibrosis and a chronic inflammatory cell infiltrate. Three patients who had complete resection of the lesion had no postoperative antifungal therapy. One of these relapsed and antifungals had to be commenced. Four patients had postoperative anti-tuberculous medication for concomitant tuberculosis or non-tuberculous mycobacterial infection.

Nineteen patients received postoperative itraconazole therapy. Among them seven had to stop itraconazole because of resistant species or adverse effects. In 16 patients voriconazole was started because of side effects or itraconazole resistance. Five patients received posaconazole postoperatively.

Patients who had intraoperative spillage of *Aspergillus* in the pleural cavity had taurolidine 2% lavage and all of those patients also had intravenous micafungin 150 mg immediately and then given daily. They subsequently had oral voriconazole, with later adjustment dependent on azole susceptibility testing.

There were no peri-operative deaths or within 30 days. Four patients (13%) died during subsequent follow-up. In the complex aspergilloma group, the actuarial survival was less (60% in 5 years) than the simple aspergilloma group (100% in 5 years) (P = 0.11 (Fisher exact)). Among the four patients who died, three of them had recurrence of aspergillosis postoperatively. One patient who had a double lung transplantation for associated cystic fibrosis died of a mycotic brain aneurysm few months following surgery, having developed ARDS after a routine surveillance bronchoscopy and lung biopsy, despite antifungal therapy [15]. The other patient who had single lung transplantation due to associated emphysema and chronic sarcoidosis died 48 months following surgery. This patient did not turn up to the follow up clinic 1 year after the operation. However, during his last postoperative follow up, the *Aspergillus* serology was negative. The other two patients who died had had a left pneumonectomy and a left upper lobectomy respectively. Both patients had postoperative recurrence and complications which included bronchopleural fistula and a persistent pleural space. They were deemed unsuitable for any further surgical intervention.

### Recurrence

Eight patients (26%) had recurrence of disease, most having prior CCPA (75%). Recurrence was documented by a combination of clinical, radiological and serological investigations. Two patients had recurrences in the pneumonectomy space immediately following surgery while the remainder had late recurrences. Among the patients who had late recurrences, two had pulmonary recurrence at a site different from the original location.

### Taurolidine MICs

All nine isolates were inhibited by taurolidine at 50 mg/L (0.1%), and 2 at 25 mg/L (0.05%), indicating a high degree of *in vitro* activity, as the solution used in patients topically is 2%.

## Discussions

CPA is more common in the developing countries due to the high prevalence of pulmonary tuberculosis [1,3]. Alongside this there has been an increase in the number of invasive aspergillosis cases in developed countries related to immunosuppression in cancer patients having intense chemotherapy and for other autoimmune diseases [16]. Like most published series [10,11] tuberculosis was the commonest underlying disease in our series.

Here we report outcomes from a wide variety of surgical procedures for chronic pulmonary aspergillosis ranging from simple wedge resection to bilateral lung transplantation in patients with pulmonary aspergillosis. Most cases of CPA are managed medically in view of the high mortality and morbidity [17], with the major exception of single aspergillomas. Simple aspergilloma cases are rare, most of our patients having CCPA. Surgery in CCPA is reserved for cases with complications or those who fail medical management. In our national centre, surgery has been reserved for patients with unilateral localised disease, failure of medical treatment and to deal with complications provided that their respiratory and performance status were adequate. Bilateral disease was not considered a contraindication per se; one patient with multiple aspergillomas underwent double lung transplantation for associated cystic fibrosis. Patients with good surgical indications, but poor nutritional status were considered for PEG feeding to improve their preoperative nutritional status. Two patients in our series had PEG feeding preoperatively. Preoperative optimisation of their respiratory status with intensive physiotherapy was routine.

Patients who presented with haemoptysis underwent bronchial artery embolization first. In patients with failed embolization, surgery was performed. Previous studies have found embolization to be ineffective or that it progressed to airway bleeding due to the existence of multiple feeder arteries from the chest wall [6,7,18]. Some authorities even recommend prophylactic excision of pulmonary aspergillomas because of risk of massive haemoptysis [6,18]. More recent angiographic series of bronchial artery embolization indicate that in ~90% of cases, control of bleeding is achieved, but that 30-50% patients rebleed by 3 years, especially if CCPA is not controlled with antifungal therapy [19].

**Figure 2:**
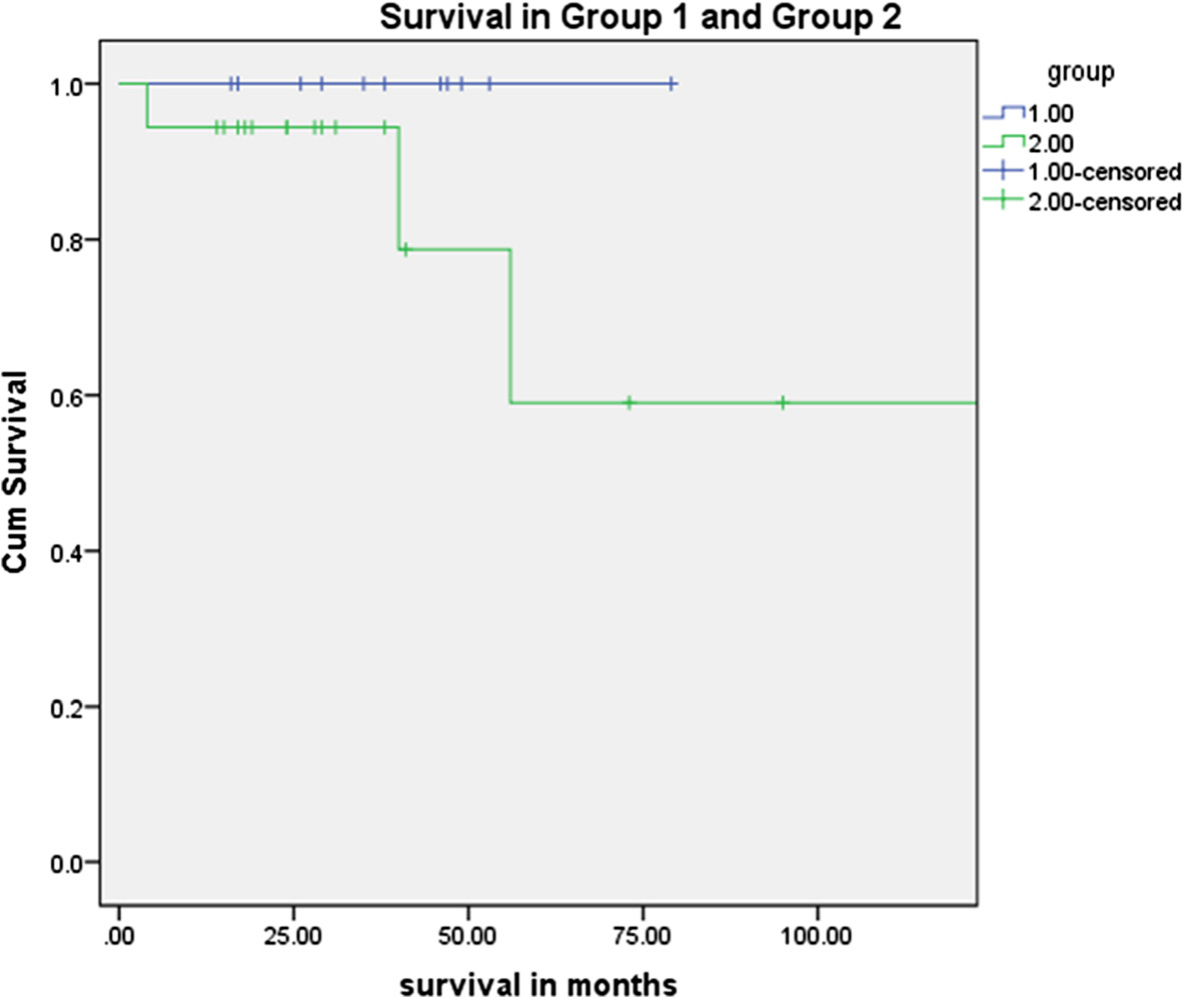
Survival curve of the patients who underwent surgery for simple and chronic pulmonary aspergillosis.

Most of our patients underwent pulmonary resection. In cases with localised unilateral disease, a wedge resection was carried out where possible to preserve lung tissue. Some authorities believe that because of the saprophytic nature of the organism parenchymal preservation is preferable provided that the rest of the underlying lung is healthy [11]. These patients who underwent a localised resection had a better outcome and in most of them postoperative antifungal therapy could be stopped. The more complex group of patients in our series who underwent surgery to deal with complications like empyema and haemoptysis had a worse outcome (Figure 2).

As others have found, patients with simple aspergillomas had better outcomes when compared to the CCPA group [9,18]. Compared to other series [9,11,20] we had better results in terms of intraoperative and 30 day mortality. We would attribute our preoperative optimisation regimes and postoperative care schedules as two leading reasons for this. Several of the studies were from Asia where patients came from rural areas with a high incidence of underlying lung disease. Results of different series for the surgical treatment of this condition have been summarized in Table 4.

**Table 4:** Results of different studies concerning surgically treated cases of Aspergilloma

**Author/year**	**Period**	**No. patients/No. operated**	**Operative mortality**	**Operative mortality in simple aspergilloma**	**Operative mortality in complex aspergilloma**
Battaglini [13] 1985	1972-1983	15/15	13.3%	0	18.1%
Daly [21] 1986	1953–1984	53/53	22.6%	4.7%	34.3%
Shirakusa [11] 1989	1979–1987	24/35	0	0	0
Massard [6] 1992	1974–1991	63/63	9.5%	0	10.0%
Regnard [22] 2000	1977-1997	87/89	5.6%	0	6.2%
Akbari [9] 2005	1985-2003	60/65	3.3%	0	4.3%
Lejay [23] 2011	1998-2009	33/33	0	0	0
Chen [20] 2012	1975-2010	256/262	1.17%	0	1.9%
Current series	1996-2011	30/33	0	0	0

One patient described here underwent completion pneumonectomy for aspergillosis in the remaining lung following a previous lobectomy for lung cancer. This patient subsequently developed bronchopleural fistula which was managed conservatively. Completion pneumonectomy has previously been reported as having a high morbidity and mortality in the presence of infection [24]. In these debilitated and often immunocompromised patients, a thoracoscopic approach has previously been described to be associated with better outcome and shorter hospital stay [25]. In our series four patients (13%) underwent thoracoscopic surgery. All of them had simple aspergillosis but two of them needed conversion to a full posterolateral thoracotomy because of intrapleural adhesions. None of the patients with CCPA were suitable for a thoracoscopic approach.

Based on our experience we have concluded that surgery should be reserved for the following group of patients: Unilateral localised disease, failure of medical treatment or to deal with complications. In addition, we have summarised the risk factors for three groups of poor outcome, namely post-operative *Aspergillus* empyema, space infection and a general poor outcome leading to death (Table 5). We cannot quantify these risks precisely and some are additive, others not.

**Table 5:** Surgical risk assessment

**Lower risk**	**Higher risk**
**Risk of *Aspergillus *empyema**	
Intrapulmonary cavity	Pleural involvement including thickening
Solid lesion	Cavitary lesion with fungal ball or fluid level
Smooth-walled cavity	Irregular or bumpy cavity surface (indicating fungal growth on surface of cavity)
Single lesion or small, localised collection of several interrelated lesions	Extensive multicavity lesion
	Prior radiotherapy to proposed surgical site
	Prior lobectomy or other thoracic surgery
**Risk of space infection**	
Localised lesion and lobectomy or segmental resection	Second lobectomy or pneumonectomy
Chest wall normal	Scoliosis or ankylosing spondylitis
	Other pleural/pulmonary disease preventing full lung mobilisation
	Immunosuppression
	Intrapleural spillage during surgery
**Risk of overall poor outcome**	
Good pulmonary function	FEV1 <1.0. L/sec
Young	Older ( >70 years)
Well nourished	Thin, low BMI or reduced albumin
No other significant comorbidities	Diabetes, other concurrent pulmonary infection (ie non-tuberculous mycobacterial or *Pseudomonas* infection)
	Other associated significant comorbidities (i.e. lymphoma, autoimmune hepatitis, organ transplantation)

The aims of antifungal therapy are to prevent *Aspergillus* empyema and to prevent recurrence of CPA post-surgery, or at least progression, if residual disease remains. Discrete *Aspergillus* nodules or simple aspergillomas that are resectable without any spillage of aspergilloma contents into the pleural space probably do not require any antifungal therapy [26]. If given, it was discontinued after surgery if a complete resection had been done. Adjuvant antifungal pharmacotherapy does not improve the results of surgical treatment for isolated pulmonary aspergillosis where a full curative resection has been carried out [26]. In the event that such spillage occurs unexpectedly, then washout of the pleural space with either amphotericin B deoxycholate or taurolidine is a reasonable, if unproven, measure to prevent *Aspergillus* empyema. *Aspergillus* empyema is a difficult to treat entity, probably requiring long term antifungal therapy and may lead to pleural fibrosis and a significant restrictive pulmonary defect if only a lobectomy or wedge resection is done.

We also recommend peri-operative antifungal therapy for patients with multi-cavity disease, in whom surgery is done because of significant haemoptysis or in an attempt to improve quality of life, or in patients in whom resection of the primary disease is likely to lead to pleural spillage. Our standard practice is to start voriconazole 2 weeks before surgery, check plasma concentrations before surgery and adjust dose if necessary and continue IV therapy through the peri-operative period. Numerous drug interactions need to be considered, including marked prolongation of sedation post-operatively. We have seen patients unconscious for 24 hours after surgery because of the excessive effect of midazolam for example. If patients are on an azole prior to surgery and there is a risk of azole resistance, we use micafungin 150 mg daily pre- and peri-operatively. In either circumstance, if no spillage occurs, we will stop antifungal therapy shortly after surgery, but give at least 2 months therapy if here has been spillage to minimize the risk of pleural aspergillosis. If patients have residual cavitary disease, we will treat post-operatively long term to prevent recurrence, just as we do in patients who do not undergo surgery [27,28].

In some patients lobectomy or pneumonectomy is possibly hazardous but a surgical necessity. Management of postoperative complications in this difficult patient group can be challenging. Creation of a modest sized fistula (the larger the better) is sometimes one approach. This is particularly so in patients with other medical problems and/or poor respiratory function, and amounts to a palliative procedure.

In patients unfit for a lobectomy drainage of the cavity (especially if there is a concurrent bacterial infection), pre-operative PEG feeding is a good first step. Following a stabilization, these patients could proceed to a definitive procedure if medically fit thereafter.

A difficult group of patients are those with CCPA who are left with a persistent space following resection. These space problems might be dealt with a pectoralis flap, modest thoracoplasty, or both. Use of a muscle flap reduces the extent of a thoracoplasty, which is helpful for later functioning of the chest. The advantage of the muscle flaps is that it often nearly fills the cavity initially, and then atrophies, leaving considerable space. Later post-op chest x rays show a small finger of muscle, but this is believed to be sufficient to keep *Aspergillus* from recolonising the cavity. Only one patient in our series underwent a thoracoplasty procedure. We planned thoracoplasty on another patient following resection; however as the patient was not fit for general anaesthesia and it couldn’t be carried out.

Postoperative follow-up continued for least 12 months, at 2 months initially and then 4 monthly, with *Aspergillus* IgG titres and a chest radiograph at a year. If there were no residual abnormalities, they were followed up for 3 years. Patients were asked to contact us on discharge if they had any new symptoms to allow us to identify expeditiously and treat patients with recurrence. *Aspergillus* IgG antibody titres usually fell very slowly following surgery, and would often level off remaining persistently elevated.

## Conclusions

Although this is a small series, it includes a wide variety of procedures that were carried out to deal with this difficult problem. We believe that proper preoperative optimisation and good postoperative care played a pivotal role in achieving these good results. The presence of a National Centre for aspergillosis and the dedicated multidisciplinary approach help maintain these high standards in our unit. We have also successfully formulated a postoperative follow up protocol which enabled early detection of recurrent disease.

We noticed more complications in the initial part of the series before designation as the National Aspergillosis Centre. Increasing experience and super specialisation has resulted in good overall outcomes. We conclude that all surgery for chronic aspergillosis should be concentrated in experienced centres. Collation of the European experience of surgical management of these cases would be a useful database. Defining strategies to select the right procedure for the right patient would be the primary aim, combined with peri-operative action plans (use of antifungals, pleural washes), actual procedural details, follow up regimes and recurrence rates.

## Abbreviations

ABPA: Allergic bronchopulmonary aspergillosis; IPA: Invasive pulmonary aspergillosis; CPA: Chronic pulmonary aspergillosis; NAC: National Aspergillosis Centre; CCPA: Chronic cavitary pulmonary aspergillosis; ARDS: Adult respiratory distress syndrome; MICs: Minimum inhibitory concentrations; PEG: Percutaneous endoscopic gastrostomy.

## Competing interests

The authors declare that they have no competing financial or non financial interests.

## Authors’ contributions

SF: Conception and design, acquisition, analysis and interpretation of data, drafting the manuscript; SM: Data collection and analysis; MD: Substantial contributions to conception and design, revising the manuscript; MK: Significant contributions in carrying out the taurilidine tests; MR: Significant contributions in carrying out the taurolidine tests; SS: Substantial contributions to revising the manuscript; MTJ: Substantial contributions to revising the manuscript; PK: Substantial contributions to revising the manuscript; RS: Substantial contributions to conception and design; revising the manuscript; DD: Critically revising the manuscript for important intellectual content. Final approval of the version to be published; RK: Conception and design, critically revising the manuscript for important intellectual content, final approval of the version to be published. All authors have read and approved the final manuscript.
